# Receiving home care forms and the risk for emergency department visits in community-dwelling Dutch older adults, a retrospective cohort study using national data

**DOI:** 10.1186/s12889-024-19305-z

**Published:** 2024-07-05

**Authors:** Oscar S Smeekes, Tim R De Boer, Robert D Van Der Mei, Bianca M Buurman, Hanna C Willems

**Affiliations:** 1 Internal Medicine, section of Geriatric Medicine, Amsterdam UMC location University of Amsterdam, Meibergdreef 9, Amsterdam, 1105 AZ the Netherlands; 2 Centrum Wiskunde & Informatica, Science Park 123, Amsterdam, the Netherlands; 3 Amsterdam UMC location Vrije Universiteit Amsterdam, Medicine for Older People, Amsterdam Public Health Research Institute, De Boelelaan 117, Amsterdam, the Netherlands

**Keywords:** Home care, Emergency department, Risk, Older adults, National data

## Abstract

**Background:**

Older adults receiving home care have a higher risk of visiting the emergency department (ED) than community-dwelling older adults not receiving home care. This may result from a higher incidence of comorbidities and reduced functional autonomy in home care recipients. Since people receive different types of home care because of their different comorbidities and autonomy profiles, it is possible that distinguishing between the form of home care can help identify subpopulations with different risks for ED visits and help develop targeted interventions. This study aimed to compare the risk of visiting the ED in older adults receiving different forms of home care with those living at home without receiving home care in a national cohort in one year.

**Methods:**

A retrospective cohort study using claims data collected in 2019 on the Dutch population aged ≥ 65 years (*N* = 3,314,440) was conducted. Participants were classified as follows: no claimed home care (NO), household help (HH), personal care (PC), HH + PC, and nursing home care at home (NHH). The primary outcome was the number of individuals that visited the ED. Secondary outcomes were the number of individuals whose home care changed, who were institutionalized, or who died. Exploratory logistic regression was applied.

**Results:**

There were 2,758,093 adults in the NO group, 131,260 in the HH group, 154,462 in the PC group, 96,526 in the HH + PC group, and 34,612 in the NHH group. More ED visits were observed in the home care groups than in the NO group, and this risk increased to more than two-fold for the PC groups. There was a significant change to a more intensive form of home care, institutionalization, or death in all groups.

**Conclusions:**

Distinguishing between the form of home care older adults receive identifies subpopulations with different risks for ED visits compared with community-dwelling older adults not receiving home care on a population level. Home care transitions are frequent and mostly involve more intensive care or death. Although older adults not receiving home care have a lower risk of ED visits, they contribute most to the absolute volume of ED visits.

**Supplementary Information:**

The online version contains supplementary material available at 10.1186/s12889-024-19305-z.

## Introduction

As the population ages with increasing comorbidity, more older adults are presenting at the emergency department (ED) [1–3]. In the Netherlands, 33% of ED visits are by older people [2], and this is expected to increase further, placing a burden on patients, society, and the health system [1, 4, 5]. Older peoples’ ED visits differ from younger people because these visits are more often a result of comorbidities and geriatric syndromes, require more time, diagnostics and hospital admittances [1]. ED’s are not designed to properly address these care needs, which leads to further health deterioration, leaves more care needs unmet, creates a need for more intense long-term care, and increases healthcare costs [6–13]. Therefore, finding ways to identify older adult populations at risk for ED visits, is essential to develop targeted interventions. Where detailed information on comorbidities, geriatric syndromes and functional autonomy help identify older adults at the individual level, it is difficult to obtain this information on a population level. The use of home care reflects the formal support needed to address these comorbidities, geriatric syndromes and loss of functional autonomy in the community and its data is often more easily accessible on a population level. However, the risk of ED visits by older adults receiving home care has not been well studied.

National data from the Dutch healthcare authority in January 2016 showed that the probability of older adults who received home care (personal care) visiting the ED was 30% compared with 9–25% in older adults not receiving home care and 17% in adults receiving nursing home care, either at home or in a nursing home [2]. A prospective cohort study followed 47 newly admitted Canadian home care recipients for 12 months and counted the number of ED visits during their home care [14]. The rate of ED visits was 3.3 per 1,000 home care days and Kaplan–Meier curves predicted that, if all recipients were followed for 5.3 months, then 50% would visit the ED. However, these results do not show whether the risk of visiting the ED differs between older adults receiving different forms of home care and how home care changes or terminates. Since older adults receive different forms of home care based on their different comorbidities and autonomy profiles, which are key contributing factors for ED visits, distinguishing between these forms of home care can help identify subpopulations with different risks. Furthermore, these insights would help us to understand why older adults visit the ED and to develop targeted interventions to prevent these visits.

The Dutch home care system provides various forms of formal home care depending on what is needed to sustain private living. Funded by government taxes and mandatory healthcare premiums, the system aims to be accessible for all Dutch inhabitants in need. When older adults experience difficulty in performing domestic tasks independently, they can apply for household help from municipalities. A municipal consultant assesses the application, conducts a home visit, and advises the municipality to what extend care is needed. This form of home care is provided by non-healthcare professionals and requires a minor individual contribution [15]. When older adults need care for medical diagnoses and functional limitations, they can apply for personal care from district nursing providers. A district nurse assesses the application, conducts a home visit and indicates to what extend care is needed. This form of home care is provided by qualified nurses and requires no contribution [16]. Household help and personal care can be combined as needed. When more intensive care at home is required due to severe medical diagnoses and functional limitations, long-term or nursing home-level support can be sought from the Care Needs Assessment Centre (CIZ), an independent governing body operating under Dutch government supervision [17]. Additional conditions include the need for around-the-clock care or supervision for the remainder of an individual’s life due to medical, psychological, or functional limitations. To apply, individuals submit a formal application explaining how they meet these criteria along with their medical file for examination by CIZ. Once approved, care administration centers, overseen by government and linked to health insurance companies, facilitate the necessary care. This form of home care is provided by qualified nurses and requires an income-dependent contribution [18]. Institutionalization becomes an option when care needs surpass what is possible at home. Most Dutch older adults live independently in private households without home care, while a minority receives home care to remain at home.

In this study, we compared the risk of visiting the ED in older adults receiving different forms of home care with those living at home without receiving home care in a national cohort. We also investigated how often older adults change their form of home care, are institutionalized, or die during a one-year follow up.

## Methods

### Study design and participants

For this retrospective cohort study, we used national aggregated data from Statistics Netherlands which covers the entire Dutch population. We retrieved claims data on home care status, institutionalization, ED visits, as well as demographics, socioeconomic parameters and medication usage. Claims data in the Netherlands, is routinely collected from both government, municipalities as well as all Dutch health insurers, and gathered in the Statistics Netherlands database [19]. We retrieved data collected in 2019 because this was before the COVID-19 pandemic and long enough after the long-term care reforms introduced in 2015. Dates of death were obtained from the death registry (see Appendix 1 and 2). Following both the guidelines of the Dutch central commission of human-bound research [20], as well as the declaration of Helsinki [21], ethics approval was not required because participation did not involve infringement of physical or psychological integrity of the participants. Furthermore, the data were anonymized and checked by Statistics Netherlands. This study was developed using the STROBE guidelines [22].

We included Dutch adults who were 65 years or older on January 1 2019, and excluded individuals who were institutionalized. We excluded individuals who were institutionalized because, despite their higher comorbidity and functional decline burden compared to community dwelling peers, they have a lower risk of emergency department visits. This is because nursing homes often manage care needs internally and do not refer to the ED [2, 23]. Because their (emergency) care needs are met differently, we excluded them from comparison.

We classified included individuals into the following five groups based on their home care status: no claimed home care (NO), household help (HH), personal care (PC), household help and personal care (HH + PC), and nursing home care at home (NHH). In order to be classified in a home care group individuals had to receive at least one day of home care in January 2019, as in concordance with earlier analyses of the Dutch healthcare authority [2]. At baseline, 3,314,440 Dutch individuals were aged 65 years and older. Of these, 2,758,093 (83.2%) were included in the NO group, 131,260 (4.0%) in the HH group, 154,462 (4.7%) in the PC group, 96,526 (2.9%) in the HH + PC group, and 34,612 (1.0%) in the NHH group. We excluded 139,487 (4.2%) individuals because they were institutionalized at baseline.

### Outcomes

Primary outcomes were the number and rate of individuals per group that visited the ED in 2019. We defined an ED visit in the same way as the Dutch health authority, including an acute presentation to any department in a hospital [24]. ED visits were scored as the number and rate of individuals that visited the ED zero, one, more than one time and four or more times at the end of follow up. Four or more visits were studied, because it is the most common used definition for frequent ED use and is to be associated with comorbidity burden [25, 26]. Secondary outcomes were the number and rate of individuals per group that received home care, were institutionalized, or died and these outcomes were recorded from February to December 2019 because of the classification method. Any changes in home care status or institutionalization were scored in the following month to avoid double registration in one group.

### Independent variables

We classified home care status as household help, personal care, household help and personal care, and nursing home care at home as provided by the Dutch home care system [15, 16, 18]. Household help, for example includes assistance with cleaning, laundry, and other household tasks. It is provided by non-healthcare personnel. It varies to what is needed, one to a few times a week, mostly a few hours a week. Personal care, for example includes medical nursing care, such as wound care, injections, and other medical procedures as well as helping with ADL. It is provided by qualified nurses (often referred to as district nurses). It varies to what is needed, one time per week to a few times a day. The national average for older adults is 4 h per week [27]. Nursing home care at home, for example includes medical nursing care and helping with disabilities of more complex and intensive in nature than personal care. It is provided by qualified nurses (often referred to as district nurses). It varies to what is needed, an hour per day to 24-hour care, mostly a few hours a day.

### Other measures

Data on age, gender, demographics, and socioeconomic status were collected in all groups on January 1 2019. We used the Statistics Netherlands definition of income class and socioeconomic status [28], with income class referring to a percentage of the social minimum household income of the household income registry and the socioeconomic status referring to a composite score (SES-score of the SES-WOA registry) of income, education, and employment. The score ranges from − 2 to 1, with a lower score corresponding to lower income, education, and/or employment. The reference SES-WOA score was 0, which is the mean score of all Dutch adults [29]. Medication use was recorded as an ATC-4 registration file that reported the number of drugs prescribed per individual.

### Statistical analysis

We compared baseline differences between the NO group and each home care group separately, as well as among the different home care groups. For dichotomous variables, we used Chi-square tests for both analyses. For continuous variables, we used t-tests to compare the NO group with the home care groups separately and one-way ANOVA to compare the different home care groups. An exploratory multivariate logistic regression analysis on the association between the different home care forms at baseline and occurring ED visits in 2019 was performed purely to correct for potential differences in age, gender and death between groups. The aim was not to make a prediction model. We limited the adjustments to correcting only for differences in age, gender, and death. Correcting for our other measures, which we believe reflect parts of the underlying mechanisms that put older adults receiving home care at risk for ED visits, would result in overcorrection [1, 30, 31]. If we were to adjust for medication use, it would partially mitigate the important effect of comorbidities on the risk of ED visits among older adults receiving home care. Two models were used. In model one, the multivariate logistic regression was adjusted for age and gender. In model two, regression was adjusted for age, gender, and death. We corrected for death because we believed it to be a significant competing risk for ED visits in the study population [32]. However, we also realize that imminent death could put older home care recipients at risk for ED visits because older adults visit the ED more often before their death [33]. This is why we tested two models. To determine changes in status during follow up in each group, the number and rate of individuals that received home care, were institutionalized, or who died were calculated monthly and visualized graphically. Statistical analysis was performed using R software [34].

## Results

### Population characteristics

The baseline characteristics are described in (Table 1). Older adults receiving home care were significantly older than those not receiving home care, were more often female, lived alone more often, had a lower SES-WOA score, had a lower income, and used more medication. Older adults receiving household help (with and without personal care) were more often female, lived alone more often, had a lower SES-WOA score, and used more medication than the other groups.

### Differences in ED visits between groups

The number and rate of ED visits, institutionalizations and deaths per home care group are described in (Table 1). All home care groups had a higher rate of ED visits than the NO group did after one year. The PC and PC + HH groups had the highest incidences of ED visits and the results between these two groups were similar. The NHH group had a higher incidence than the HH group, but a lower incidence than the PC groups. The NO group had the lowest rate of individuals that visited the ED (13.8%), but contributed most to the total number of individuals visiting the ED (381,523) because of its larger size. All home care groups had a relatively higher rate of more than one ED visit, and especially four or more visits, compared to the NO group. These rates were highest in the PC groups. All home care groups had a higher institutionalization and death rate than the NO group did, especially the NHH group.

**Table 1:** Population characteristics of Dutch adults aged 65 years and older on January 1 2019, their ED visits, institutionalizations and deaths at the end of follow up

Characteristics	NO	HH	PC	HH + PC	NHH	*P*-values^a, b,c, d,e^
**N**	2,758,093	131,260	154,462	96,526	34,612	
**Age (mean in years) [SD]**	73.1 [6.2]	78.9 [7.4]	81.1 [7.7]	82.3 [7.7]	83.2 [7.8]	*P* < 0.001^a, b,c, d,e^
**Gender (female %)**	51	74	58	75	65	*P* < 0.001 ^a, b,c, d,e^
**Lives alone (%)**	26.8	73.7	48.2	77.5	42.6	*P* < 0.001 ^a, b,c, d,e^
**SES-WOA score** ^**1**^ **(mean) [SD]**	-0.1 [0.5]	-0.6 [0.5]	-0.3 [0.5]	-0.6 [0.5]	-0.3 [0.5]	*P* < 0.001 ^a, b,c, d,e^
**Income** ^**2**^ **(mean) [SD]**	238 [128.0]	143.9 [54.2]	188.4 [97.7]	143.2 [57.0]	178.2 [92.6]	*P* < 0.001 ^a, b,c, d,e^
**Medication** ^**3**^ **(mean)** **[SD]**	5.5 [4.2]	8.8 [4.7]	9.9 [5.0]	10.9 [5.0]	9 [4.8]	*P* < 0.001 ^a, b,c, d,e^
**Number of individuals that visited the ED in 2019 (%)**	381,523 (13.8%)	31,431 (24.0%)	53,531 (34.7%)	33,866 (35.1%)	10,902 (31.5%)	*P* < 0.001 ^a, b,c, d,e^
**1 ED visit in 2019 (%)**	254,135 (9.2%)	19,040 (14.5%)	29,744 (19.3%)	18,614 (19.3%)	6,384 (18.4%)	*P* < 0.001 ^a, b,c, d,e^
**> 1 ED visit in 2019 (%)**	127,388 (4.6%)	12,391 (9.4%)	23,787 (15.4%)	15,252 (15.8%)	4,518 (13.1%)	*P* < 0.001 ^a, b,c, d,e^
**4 or more ED visits in 2019 (%)**	21,732 (0.8%)	2,307 (1.8%)	5,159 (3.3%)	3,200 (3.3%)	765 (2.2%)	*P* < 0.001 ^a, b,c, d,e^
**Number of individuals that were institutionalized in 2019** ^**4**^ **(%)**	7,663 (0.3%)	1,557 (1.2%)	8,793 (5.7%)	6,603 (6.8%)	9,168 (26.5%)	*P* < 0.001 ^a, b,c, d,e^
**Number of individuals that died in 2019 (%)**	51,583 (1.9%)	6,199 (4.7%)	25,141 (16.3%)	13,158 (13.6%)	7,206 (20.8%)	*P* < 0.001 ^a, b,c, d,e^

*Abbreviations: NO = no claimed home care, HH = household help, PC = personal care, HH + PC = household help and personal care, NHH = nursing home care at home.*^***1***^*The SES-WOA score is a mean composite score of income, education, and employment and ranges from − 2 to 1, with a lower score corresponding to a lower income, education, and/or employment. The reference SES-WOA score is 0, which is the mean score of all Dutch adults* [29] ^*2*^*Income is reported as a percentage of the social minimum, where 100 corresponds to a mean income equal to social minimum.*^*3*^*Medication is the mean cumulative use over a year of distinct prescribed drugs based on a 2019 ATC-4 code.*^*4*^*As a result of the used classification method and of scoring change in care the following month, recording of change in care was limited to the period of February to December 2019, eleven months instead of twelve and thereby influenced the end results.*^*a*^*t-tests (for continuous variables) or Chi-square tests (for dichotomous variables) to compare the NO group with HH.*^*b*^*t-tests (for continuous variables) or Chi-square tests (for dichotomous variables) to compare the NO group with PC.*^*c*^*t-tests (for continuous variables) or Chi-square tests (for dichotomous variables) to compare the NO group with HH + PC.*^*d*^*t-tests (for continuous variables) or Chi-square tests (for dichotomous variables) to compare the NO group with NHH.*^*e*^*one-way ANOVA (for continuous variables) or Chi-square tests (for dichotomous variables) to compare the different home care groups. All P-values were < 0.001, indicating significant differences.*

### Exploring the association between home care forms and ED visits, correcting for differences in age, gender and death

(Table 2) shows the results of the exploratory logistic regression models. All home care forms were significantly associated with a higher risk for ED visits, with the highest risk in the HH and NHH groups.

**Table 2:** Exploratory odds ratios for an ED visit per home care group

Group	Odds ratio model one, correcting for age & gender (CI 95%)	Odds ratio model two, correcting for age, gender & death (CI 95%)
**NO**	1	1
**HH**	1.67 (1.63–1.67)	1.63 (1.60–1.65)
**PC**	2.56 (2.51–2.61)	2.08 (2.05–2.10)
**HH + PC**	2.57 (2.51–2.61)	2.20 (2.17–2.24)
**NHH**	2.11 (2.08–2.16)	1.55 (1.51–1.60)

*Abbreviations: NO = no claimed home care, HH = household help, PC = personal care, HH + PC = household help and personal care, NHH = nursing home care at home.*

### The dynamics of home care during follow up

**Fig. 1:**
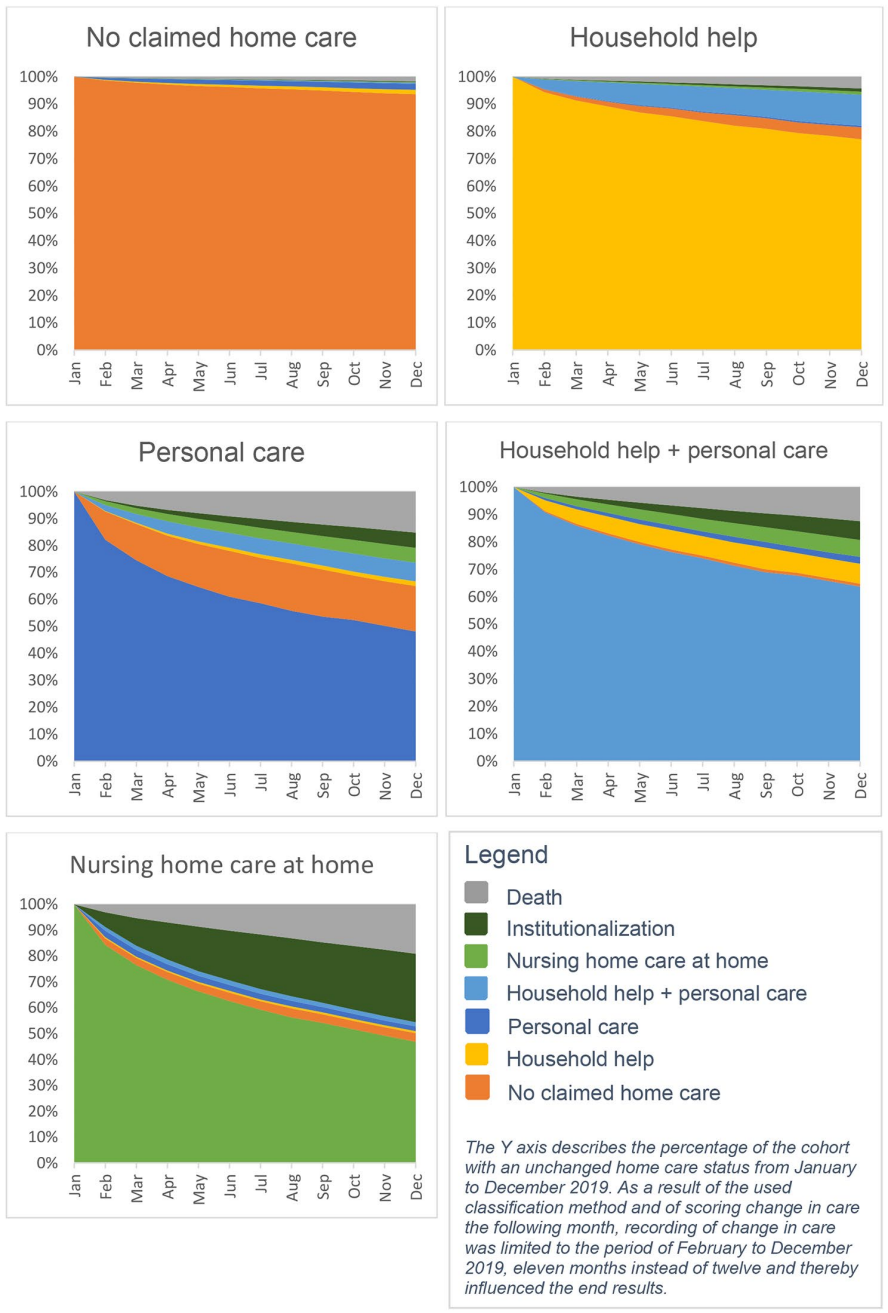
The dynamics of home care, institutionalization, and death in 2019 in each group

(Fig. 1) describes the monthly results for home care, institutionalization, and death from February to December 2019. In the NO group, 6.4% (*n* = 175,950) changed in home care status (including 1.6% to household help, 2.2% to personal care, 0.4% to household help and personal care, 0.2% to nursing home care at home, 0.3% to institutionalization and 1.9% died). In the HH group, 22.9% (*n* = 30,053) changed in home care status (including 4.4% to no claimed home care, 0.6% to personal care, 11.3% to household help and personal care, 1.1% to nursing home care at home, 1.2% to institutionalization and 4.7% died). In the PC group, 51.9% (*n* = 80,115) changed in home care status (including 16.8% to no claimed home care, 1.7% to household help, 7.0% to household help and personal care, 5.4% to nursing home care at home, 5.7% to institutionalization and 16.3% died). In the PC + HH group, home care status changed in 36.3% (*n* = 35,026) of individuals (including 1.1% to no claimed home care, 7.3% to household help, 2.5% to personal care, 6.1% to nursing home care at home, 6.8% to institutionalization and 13.6% died). In the NHH group, home care status changed in 53.1% (*n* = 18,394) of individuals (3.3% to no claimed home care, 0.8% to household help, 1.8% to personal care, 1.6% to household help and personal care, 26.5% to institutionalization and 20.8% died). It seems unlikely that individuals of the NHH group that changed to no claimed home care (*n* = 1,158) did not require any home care because they had a previous 24-hour nursing home care at home indication. A possible explanation could be that these individuals changed to privately funded home care.

## Discussion

This study shows that older adults receiving home care have a higher risk of visiting the ED than individuals without home care do and this risk increases to more than two-fold for those receiving nurse-based, medically and functionally indicated personal care. Furthermore, home care status changed considerably over the one-year follow-up period, mostly to a more intense form of home care, institutionalization, or death. Moreover, the largest proportion of ED visits came from older adults not receiving formal support at home, despite their lower risk of an ED visit.

Earlier studies have identified an increased risk of visiting the ED in older adults receiving home care compared with those not receiving formal support [2, 14, 23]. Our findings confirm these earlier observations [2, 14, 23] and further demonstrate that the ED visit risk is increased in recipients of all forms of home care, even household help alone. A detailed comparison on risks is difficult to make with earlier literature, because these studies did not differentiate between home care forms and used different outcomes. We observed the highest risk in individuals receiving nurse-based, medically and functionally indicated personal care. Age, gender, and death differed significantly between the home care groups and NO group, but the higher risk of visiting the ED in home care recipients remained after correcting for these factors. The difference in ED visit risk between those not receiving home care and those who did was greatest for more frequent visits. This supports the hypothesis that chronic comorbidities play a key role in the ED visit risk for home care recipients [25, 26]. Singular visits often result from an acute care need that is effectively addressed, while recurrent visits are more often due to chronic conditions that are insufficiently addressed [25, 26].

These observations could be due to higher frailty in home care recipients. We base this hypothesis on the following arguments. First, the indication for home care is based on an individual’s ability to live independently, so reflects a person’s functionality, which is one of the domains used to diagnose frailty [15, 16, 18]. Compared with individuals receiving no home care, home care recipients are probably frailer, varying from mildly frail (limited functional decline) in individuals receiving household help, intermediately frail (more distinct functional decline) in individuals receiving personal care, to severely frail (obvious functional restrictions) in individuals receiving nursing home care at home. Second, frailty has been identified as an important driver for ED visits and acute hospitalization in recipients of personal care [35, 36]. However, our results showed a lower ED risk in the NHH group, which is probably the most frail group. We have two explanations for this. First, recipients of nursing home care at home in the Netherlands are often admitted to nursing homes instead of EDs when acute care demands arise, because they require 24-hour care or nursing home care [37]. Second, as demonstrated in Fig. 1, a large portion of this group was institutionalized during follow up. Further research is needed to test this hypothesis.

We found that most older Dutch adults who visited the ED live independently without any formal support. These results are in line with those of previous reports on older adults visiting the ED [1–3, 5, 30, 31, 35, 38]. Older adults living independently without any formal support are less frail, present less frequently, have fewer geriatric syndromes, and fewer unstable comorbidities, so present more often with acute complaints [23, 35]. These differences may require different preventive approaches. Therefore, to fully address the increasing volume of older adults at the ED, future research should focus on how to prevent ED visits in older adults not receiving home care.

This is the first study to provide a month-to-month insight into all forms of home care on a national level, adding to similar longitudinal results of specific subgroups [2, 39–41]. Changes in home care were observed in all groups during the follow-up period, and most of these changes involved more intense care, institutionalization, or death. Our findings in individuals receiving personal care illustrate the value of a monthly follow up. Older adults who transitioned to the NO group typically did so within 2 months, while those who did not recover in the first few months gradually needed more intense home care, or were institutionalized or died. Thereby, we identified two different subgroups. We hypothesize that the subgroup that recovers quickly has a temporary need for support and is temporarily frail, while the other group gradually needs more intense care because of increasing frailty. This hypothesis is endorsed by earlier studies [39, 41–46].

### Limitations

Despite the valuable insights the used database provides, there are several limitations that need to be taken into account. First, the absence of informal care data, privately funded home care, and partially unclaimed home care limits insight on provided help at home. We think the effect of the latter two is limited because home care in the Netherlands is primarily publicly funded and aims to be accessible to all in need. Second, the database does not provide accurate data on the intensity of home care received, in particular on how many hours of home care were provided and what for they were used. Therefore, we did not include these in our analysis. As a result, people receiving many hours or a high intensity of a form of home care are classified the same as people receiving a few hours or a low intensity, and the group’s average result is calculated. Therefore, our results are limited to the group average. However, the intensity of care provided is to a certain extent reflected by the different home care forms because they are part of the eligibility criteria form these forms as described in the introduction and method section. Third, the database currently does not include useful data on the number and type of comorbidities as well as geriatric syndromes, limiting our comparison of underlying risk factors for ED visits between groups, correlating them to the ED visits and assessing whether a visit was avoidable. Fourth, the database currently does not include useful data on primary care visits, which limits our understanding of whether attempts have been made to prevent developing acute care demands leading to ED visits, and thus the quality of this care. We think the effects of missing primary care data on the results of this study are limited because primary care in the Netherlands is equally accessible to all the studied groups. Fifth, this study has been conducted studying a Dutch national sample receiving Dutch home health care. We have done our best to characterize the sample and home health care system for assessment of generalizability. Similar populations and systems include Northern Europe and Canada [11, 47]. Whereas the US emphasizes on privately funded home care, and Asia on family-centered informal care [48, 49]. This should be taken in to account when assessing generalizability.

Future research on the risk of older people visiting the ED should investigate the mechanisms that underlie this risk in older adults receiving different forms of home care, further test the hypotheses and limitations of this study, and develop targeted interventions. Prevention strategies have potential in older adults receiving home care because these individuals are more likely to visit the ED and are already being visited by health or social care professionals on a daily basis. Interventions that target the known risk factors in home care recipients, such as early detection and treatment of geriatric syndromes and comorbidities, could be a first step. Because the challenges and principles for home care across most Western countries are similar, generalizability of interventions seems fruitful [11, 23].

## Conclusion

This study shows, on a national level, that community-dwelling older adults receiving home care have an increased risk of visiting the ED and that this risk differs between older adults receiving different forms of home care. Older adults receiving nurse-based, medically and functionally indicated personal care are most at risk. They face a more than twofold risk compared to their peers not receiving home care, in particular for more frequent visits. These older adults are visited daily by professionals, so there is potential for targeted interventions in the future. This requires further investigation into the causal factors contributing to the risk of ED visits among home care recipients, particularly comorbidities, geriatric syndromes, and the quality of both primary and home care. Furthermore, when assessing the risk of visiting the ED in older adults, one should take into account that home care changes considerably over a one-year period, mostly to more intense care, institutionalization, or death. Moreover, older adults not receiving home care contribute most to the national volume of ED visits by older adults, despite their lower ED visit risk and may need a different preventive approach.

### Electronic supplementary material

Below is the link to the electronic supplementary material.

Supplementary Material 1

## Data Availability

Restrictions apply to the availability of the original data, which were used under approval of the current study, and so are not publicly available. Approval can be obtained from Statistics Netherlands. However, all output and analyses of the current study are reported in the article.

## Abbreviations

ED: Emergency department
NO: No claimed home care
HH: Household help
PC: Personal care
NHH: Nursing home care at home
SES-WOA: Score socio economic status-wealth education and employment history score
