# Supplementary material for: Receiving home care forms and the risk for emergency department visits in community-dwelling Dutch older adults, a retrospective cohort study using national data
Source: BMC Public Health. 2024 Jul 5;24:1792. doi: 10.1186/s12889-024-19305-z (PMC11225288; doi:10.1186/s12889-024-19305-z)
Supplement: Supplementary file 1 — Supplementary Material 1 [file 12889_2024_19305_MOESM1_ESM.docx]

**Appendices 1-2 list of registries and codes,** Receiving home care forms and the risk for emergency department visits in community-dwelling Dutch older adults, a retrospective cohort study using national data

1. Used registries
2. Used codes
3. **Used registries**

The following databases of CBS were used for this paper (description of data included in these registries is in Dutch):

- GBAHUISHOUDENBUS

<https://www.cbs.nl/nl-nl/onze-diensten/maatwerk-en-microdata/microdata-zelf-onderzoek-doen/microdatabestanden/gbahuishoudensbus-huishoudenskenmerken>

- MEDICIJNTAB

<https://www.cbs.nl/nl-nl/onze-diensten/maatwerk-en-microdata/microdata-zelf-onderzoek-doen/microdatabestanden/medicijntab-geneesmiddelen-op-atc-code--4-->

- INHATAB

<https://www.cbs.nl/nl-nl/onze-diensten/maatwerk-en-microdata/microdata-zelf-onderzoek-doen/microdatabestanden/inhatab-inkomen-van-huishoudens>

- KOPPELPERSOONHUISHOUDEN
- GBAOVERLIJDENTAB

<https://www.cbs.nl/nl-nl/onze-diensten/maatwerk-en-microdata/microdata-zelf-onderzoek-doen/microdatabestanden/gbaoverlijdentab-datum-van-overlijden-van-personen-ingeschreven-in-het-gba>

- SESWOA

<https://www.cbs.nl/nl-nl/onze-diensten/maatwerk-en-microdata/microdata-zelf-onderzoek-doen/microdatabestanden/seswoa-sociaaleconomische-statusscores-huishoudens>

- WLZINTAB

<https://www.cbs.nl/nl-nl/onze-diensten/maatwerk-en-microdata/microdata-zelf-onderzoek-doen/microdatabestanden/wlzzintab-personen-metgebruik-wlz-zorg-in-natura>

- GEBWMOTAB

<https://www.cbs.nl/nl-nl/onze-diensten/maatwerk-en-microdata/microdata-zelf-onderzoek-doen/microdatabestanden/gebwmotab-personen-met-wmo-maatwerkvoorzieningen>

- ZVWWVPTAB

<https://www.cbs.nl/nl-nl/onze-diensten/maatwerk-en-microdata/microdata-zelf-onderzoek-doen/microdatabestanden/zvwwvptab-personen-met-gebruik-van-zvw-wijkverpleging>

- GBAPERSOONKTAB

<https://www.cbs.nl/nl-nl/onze-diensten/maatwerk-en-microdata/microdata-zelf-onderzoek-doen/microdatabestanden/gbapersoonktab-persoonskenmerken-beperkt-in-de-brp>

- MSZZorgactiviteitenVEKTTAB

<https://www.cbs.nl/nl-nl/onze-diensten/maatwerk-en-microdata/microdata-zelf-onderzoek-doen/microdatabestanden/mszzorgactiviteitenvekttab-zorgactiviteiten-diagnose>

1. **Used codes**

- Household help (WMO codes): 006,007,100,101,102,103,104,105,107,711
- Personal care (ZVW codes): 1, 2, 3 ,4
- Nursing home care at home (WLZ codes): 2 and 3
- ED codes: 19015 (ED admittance) and 19016 (acute admittance outside ED)
- Institutionalization (WLZ codes): 1, 11 and 12.

**References**

1 Aminzadeh F, Dalziel WB. Older adults in the emergency department: A systematic review of patterns of use, adverse outcomes, and effectiveness of interventions. *Ann Emerg Med* 2002;**39**:238–47.

2 O’Cathain A, Knowles E, Turner J, Maheswaran R, Goodacre S, Hirst E, *et al.* Explaining variation in emergency admissions: a mixed-methods study of emergency and urgent care systems. *Health Services and Delivery Research* 2014;**2**:1–126.

3 Gray LC, Peel NM, Costa AP, Burkett E, Dey AB, Jonsson P v., *et al.* Profiles of older patients in the emergency department: findings from the interRAI Multinational Emergency Department Study. *Ann Emerg Med* 2013;**62**:467–74.

4 Statistics Netherlands. Dutch population development, death in specific. https://www.cbs.nl/nl-nl/visualisaties/dashboard-bevolking/bevolkingsgroei/overlijden (last accessed 5th June 2023). 2023.https://www.cbs.nl/nl-nl/visualisaties/dashboard-bevolking/bevolkingsgroei/overlijden (accessed 17 Mar2023).

5 Smith AK, McCarthy E, Weber E, Cenzer IS, Boscardin J, Fisher J, *et al.* Half of older Americans seen in emergency department in last month of life; most admitted to hospital, and many die there. *Health Aff (Millwood)* 2012;**31**:1277–85.
